# Real-Time High Resolution 3D Imaging of the Lyme Disease Spirochete Adhering to and Escaping from the Vasculature of a Living Host

**DOI:** 10.1371/journal.ppat.1000090

**Published:** 2008-06-20

**Authors:** Tara J. Moriarty, M. Ursula Norman, Pina Colarusso, Troy Bankhead, Paul Kubes, George Chaconas

**Affiliations:** 1 Department of Biochemistry & Molecular Biology, University of Calgary, Calgary, Alberta, Canada; 2 Department of Microbiology & Infectious Diseases, University of Calgary, Calgary, Alberta, Canada; 3 Department of Physiology & Biophysics, University of Calgary, Calgary, Alberta, Canada; Medical College of Wisconsin, United States of America

## Abstract

Pathogenic spirochetes are bacteria that cause a number of emerging and re-emerging diseases worldwide, including syphilis, leptospirosis, relapsing fever, and Lyme borreliosis. They navigate efficiently through dense extracellular matrix and cross the blood–brain barrier by unknown mechanisms. Due to their slender morphology, spirochetes are difficult to visualize by standard light microscopy, impeding studies of their behavior *in situ*. We engineered a fluorescent infectious strain of *Borrelia burgdorferi*, the Lyme disease pathogen, which expressed green fluorescent protein (GFP). Real-time 3D and 4D quantitative analysis of fluorescent spirochete dissemination from the microvasculature of living mice at high resolution revealed that dissemination was a multi-stage process that included transient tethering-type associations, short-term dragging interactions, and stationary adhesion. Stationary adhesions and extravasating spirochetes were most commonly observed at endothelial junctions, and translational motility of spirochetes appeared to play an integral role in transendothelial migration. To our knowledge, this is the first report of high resolution 3D and 4D visualization of dissemination of a bacterial pathogen in a living mammalian host, and provides the first direct insight into spirochete dissemination *in vivo*.

## Author Summary

Pathogenic spirochetes are bacteria that cause a number of emerging and re-emerging diseases worldwide, including syphilis, leptospirosis, relapsing fever, and Lyme disease. They exhibit an unusual form of motility and can infect many different tissues; however, the mechanism by which they disseminate from the blood to target sites is unknown. Direct visualization of bacterial pathogens at the single cell level in living hosts is an important goal of microbiology, since this approach is likely to yield critical insight into disease processes. We engineered a fluorescent strain of *Borrelia burgdorferi*, a Lyme disease pathogen, and used conventional and spinning disk confocal intravital microscopy to directly visualize these bacteria in real time and 3D in living mice. We found that spirochete interaction with and dissemination out of the vasculature was a multi-stage process of unexpected complexity and that spirochete movement appeared to play an integral role in dissemination. To our knowledge, this is the first report of high resolution 3D visualization of dissemination of a bacterial pathogen in a living mammalian host, and provides the first direct insight into spirochete dissemination *in vivo*.

## Introduction

Pathogenic spirochetes are bacteria that cause a number of emerging and re-emerging diseases worldwide, including syphilis, leptospirosis, relapsing fever and Lyme borreliosis [1]–[6]. Many clinically-important spirochetes cross the blood-brain barrier and exhibit an unusual form of motility that is predicted to permit efficient movement through dense extracellular matrix in host tissues [6]–[9]. Spirochetes of the *Borrelia burgdorferi sensu lato* species complex are the causative agents of Lyme borreliosis [1],[10]. *B. burgdorferi* are transmitted to the skin of mammalian hosts through the bite of an infected tick. Subsequently they enter the vascular circulation and disseminate hematogenously to multiple tissues by unknown mechanisms. Untreated Lyme borreliosis can result in arthritis, carditis and neurological complications.

*B. burgdorferi* and other spirochetes interact with endothelial cells under static conditions *in vitro* [11]–[13]. However, spirochete-vascular interactions have never been examined in the host itself, or under the fluid shear forces that are present at dissemination sites. Indeed, host-pathogen interactions under shear stress conditions are still poorly understood for most bacterial pathogens that invade or disseminate in the mucosa or blood vessels, despite the importance of shear forces present in these environments.

Early studies with cultured endothelial cells found that treatments or mutations that rendered *B. burgdorferi* non-motile impaired invasion but not interaction [14]–[17], suggesting that the spirochete's ability to bore through dense tissues using translational motility might be important for vascular invasion. However, all previous investigations of *B. burgdorferi* dissemination were performed *ex vivo* in the absence of shear stress, using endothelial cell monolayers incubated with *B. burgdorferi* for periods as long as 24 hours, and employed non-dynamic visualization techniques such as electron microscopy which precluded observation of spirochete movement [14], [15], [17]–[19]. Conflicting reports found that extravasating *B. burgdorferi* were localized exclusively in either endothelial junctions or cells [14],[15],[18]. The role of host cells in transmigration was also controversial, because electron microscopy studies revealed no unambiguous evidence of endocytosis, and since the host microfilament toxin cytochalasin D did not inhibit spirochete internalization [14],[15]. Thus, the mechanism of *B. burgdorferi* dissemination in mammalian hosts remains a mystery.

It has been challenging to study host-spirochete interactions in a living host because their slender (<1 µm) morphology makes them difficult to visualize by standard light microscopy. Direct observation of Lyme borreliosis spirochete interactions with mammalian cells has been limited to cell culture models or host tissues removed from their native context. Several green fluorescent protein (GFP) alleles have been expressed in *B. burgdorferi*, usually in the context of reporter constructs used to monitor gene transcription and plasmid maintenance in spirochetes grown in culture [20]–[23]. Recently, a GFP reporter construct was used to monitor gene expression during *B. burgdorferi* infection, in tissues that were excised from the host before visualization [24]; however, detection of the GFP allele used in this study required relatively long, two second exposure times [23].

Intravital microscopy (IVM) is a powerful tool for studying the cellular dynamics of the immune and cardiovascular systems and tumor metastasis in the context of a living organism [25],[26]. It has also been used to visualize tissue localization dynamics of bacterial pathogens in vertebrate hosts [27],[28]. However, the small size of most pathogens and the spatial resolution limits of conventional epifluorescence IVM have impeded analysis of live host-pathogen interactions at the single cell level. The application of spinning disk confocal microscopy in an intravital setting finally enabled real-time visualization of transmission of malaria parasites to a living host [29], but unambiguous analysis of dissemination by smaller pathogens such as bacteria requires the ability to perform three-dimensional (3D) microscopy *in vivo*. In the current study we report the first use of spinning disk confocal microscopy to visualize dynamic host-pathogen interactions in three and four dimensions, revealing many aspects of the *B. burgdorferi* dissemination process that have not been previously observed, *in vitro* or *in vivo*. The ability to examine bacterial pathogenesis over time and in the three-dimensional space of living hosts will greatly enhance our understanding of many infectious diseases.

## Results

### Engineering of fluorescent infectious *B. burgdorferi*

**Figure 1:**
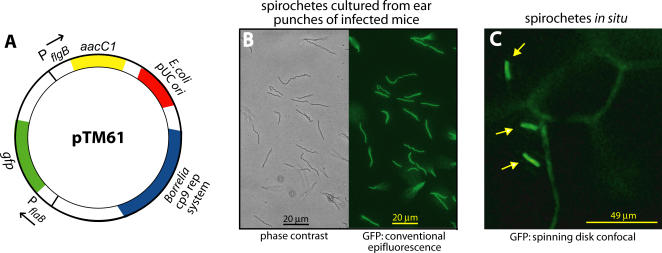
Engineering and *in situ* visualization of infectious fluorescent *B. burgdorferi*. A) Schematic of plasmid pTM61, which was used to constitutively express green fluorescent protein (GFP) under the control of the *B. burgdorferi flaB* promoter (P*_flaB_*). The construct also carries determinants for replication in both *E. coli* and *B. burgdorferi*, as well as a gentamycin resistance (*aacC1)* cassette. B) Phase contrast and epifluorescent visualization of spirochetes cultured from mouse ears. Ear punches obtained from infected C3H/HeN mice 13 days after inoculation were cultured in BSK-II medium in the absence of gentamycin selection before visualization. C) Visualization of live fluorescent *B. burgdorferi* in a living mouse ear by spinning disk confocal IVM. The spirochetes, indicated by yellow arrows, were observed four weeks after intraperitoneal inoculation of a C57 BL/6 mouse. Blood vessels were visualized by jugular vein injection of FITC-labeled albumin. Video footage of fluorescent *B. burgdorferi* moving in the ear of a living C57 mouse is presented in Video S1.

*B. burgdorferi* are difficult to modify genetically, and transformation with recombinant constructs often results in the loss of plasmids that are required for infectivity in the mouse [30]. We were able to engineer infectious and non-infectious *B. burgdorferi* expressing a highly fluorescent GFP allele optimized for bacterial expression [20],[31] (**Fig. 1A**); this allele is distinct from the *egfp* [21],[23] and *gfpmut1* [21],[23] alleles that have also been expressed in *B. burgdorferi*. The resulting infectious (GCB726) and non-infectious (GCB705) strains displayed similar levels of GFP fluorescence, which could be detected with very short exposure times of less than 100 ms with a conventional epifluorescence microscope. The infectious strain contained the full complement of *B. burgdorferi* plasmids required for infectivity (see **Materials and Methods**).

To confirm that fluorescent strain GCB726 was infectious, and to determine if GFP expression could be stably maintained without antibiotic selection in infected murine hosts, two C3H/HeN and two C57 BL/6 mice were inoculated with 5.5×10^4^ fluorescent spirochetes. Two ear punches were collected for each mouse 13 days (C3H mice) or 28 days post-infection (C57 mice), and cultured in *B. burgdorferi* growth medium with or without gentamycin selection. Spirochetes were recovered from all ear punches in both the presence and absence of antibiotic, confirming that strain GCB726 was infectious in both C3H and C57 mice. Furthermore, 98.7 −/+ 1.3% of the *B. burgdorferi* cultivated in the absence of gentamycin retained robust levels of GFP expression (**Fig. 1B** **)**, indicating that the GFP-expressing plasmid was stably maintained in the context of the murine host.

### Real-time visualization of fluorescent spirochetes at the single cell level in a living murine host

We next investigated whether fluorescent *B. burgdorferi* could be exploited for real-time studies of host-spirochete interactions using intravital microscopy (IVM). Fluorescent infectious spirochetes were observed *in situ* in the ears of living C3H mice 20 and 27 days post-infection and in the ears of C57 mice 28 days after infection, using both conventional epifluorescence and spinning disk confocal IVM. Consistent with a previous report that *B. burgdorferi* localize to the perivascular connective tissue [32], fluorescent *B. burgdorferi* were observed outside, but usually close to blood vessels, and frequently translated back and forth repetitively over a relatively lengthy distance (**Fig. 1C** and **Video S1**). *In situ*, *B. burgdorferi* exhibited all of the translational (running) and non-translational (flexing) modes of movement that are characteristically observed in culture medium [7],[33].

Although spirochetes traveled more slowly and reversed directions more frequently when passing around visible obstacles such as blood vessels, they could achieve speeds of up to 4.0 µm/s in the ear, a speed which is very similar to the previously reported 4.25 µm/s *in vitro* rate [34]. Interestingly, although *B. burgdorferi in situ* often reversed their direction of movement every few seconds, which is typical of translational motility *in vitro* [35], they also exhibited sustained unidirectional movement for periods as long as 40 seconds.

### A real-time experimental model system for studying *B. burgdorferi* behavior in the host microvasculature

In order to investigate the behavior of spirochetes in the host microvasculature, fluorescent *B. burgdorferi* were injected directly into the bloodstream of C57 mice via the jugular or femoral veins, and were visualized in real-time using both conventional epifluorescence and spinning disk confocal IVM. Prior to inoculation, fluorescent spirochetes were cultured for 48 hours in the presence of 1% mouse blood to promote adaptation to the host environment, since growth in blood is known to regulate the expression of many *B. burgdorferi* genes [36].

**Figure 2:**
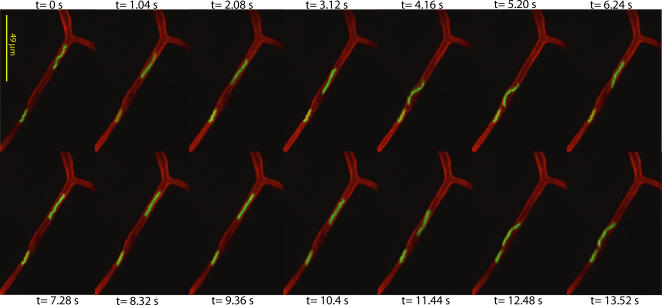
Real-time visualization of *B. burgdorferi* in the microvasculature of a living murine host. A time-lapse microscopy series of two GFP-expressing *B. burgdorferi* in the capillary of the skin of a living C57 mouse is shown. The top spirochete is motile while the bottom spirochete is stationary. Time (t) in seconds (s) is shown above or below each micrograph in the series. Images were obtained by spinning disk confocal IVM after jugular vein injection of fluorescent *B. burgdorferi.* Endothelial cells were labeled with Alexa Fluor 555-conjugated antibody to PECAM-1, which is expressed on endothelial cells and concentrates at intercellular junctions [38]. Direction of blood flow in this vessel is unknown. The video from which these micrographs were extracted is presented as Video S2. Video S3 is a representative video of spirochete interactions in a postcapillary venule.

Vascular interactions were analyzed in flank skin, where the best optical clarity was obtained (see **Fig. 2** and **Video S2**). The relevance of this site as a target for *B. burgdorferi* dissemination was confirmed by recovery of spirochetes from cultures of skin taken from mice 28 days post-infection. Fluorescent *B. burgdorferi* maintained a stable density in the bloodstream for longer than four hours after injection, but interactions with the microvasculature were analyzed between 5 and 45 minutes after injection during which time endothelial activation was not observed and spirochete titers and rates of interaction were stable (see **Text S1** for data and discussion related to the lack of endothelial activation).

Examination of interactions between fluorescent *B. burgdorferi* and the microvasculature in more than 40 mice yielded several general observations. First, at similar blood densities, non-infectious fluorescent spirochetes did not associate with blood vessels, though they remained in the circulation, indicating that vascular interactions were not an artifact caused by high blood titers or by mechanical impediments to cell flow.

Second, infectious spirochetes associated with capillaries, postcapillary venules and larger veins, but not with arterioles (**Fig. 2**, **Videos S2, S3** and **S4**). Vessel identity was determined by measurement of vessel diameters and by observation of blood flow patterns in the immediate vascular network (convergence indicates venules while divergence identifies arterioles). The observation that spirochetes did not interact with the lumenal surface of arterioles differs from the conclusions derived from a previous report of mice infected intradermally with *B. burgdorferi*, which found that after several weeks of infection spirochetes were preferentially localized to the walls of arterial vessels [32]. One possible explanation for this discrepancy is that in the previous study spirochetes might have migrated into the walls of arterial vessels from extravascular tissues, an event that is unlikely to have occurred during the short time frame of our experiments. Additionally, colonization of the connective tissue-rich walls of arteries could be promoted by bacterial adaptation to the host environment during longterm infections. It was unlikely that the inability of *B. burgdorferi* to interact with arterioles in the time frame of our experiments was due to differences in expression of host cell ligands in arterial and venous vessels, since spirochetes readily associated with the arterial endothelium under conditions of reduced blood flow. Therefore, reduced spirochete interactions in arterioles may have resulted from the elevated shear forces present in these vessels.

Third, interacting spirochetes in capillaries sometimes moved back and forth with and against the direction of blood flow (**Fig. 2**, **Video S2**). In contrast *B. burgdorferi* usually moved with the direction of flow in venules and veins where blood flow was more rapid, but moved freely in multiple directions under conditions of reduced blood flow. From these qualitative observations we infer that spirochete interactions with the host microvasculature are strongly affected by blood flow.

Finally, *B. burgdorferi* grown in the presence or absence of blood did not exhibit significant differences in the total number or type of interactions. This suggested that blood-stimulated gene expression in *B. burgdorferi* [36] was not a requirement for microvascular interaction.

### Identification of three types of *B. burgdorferi* interactions in the postcapillary venules of living mice

**Figure 3:**
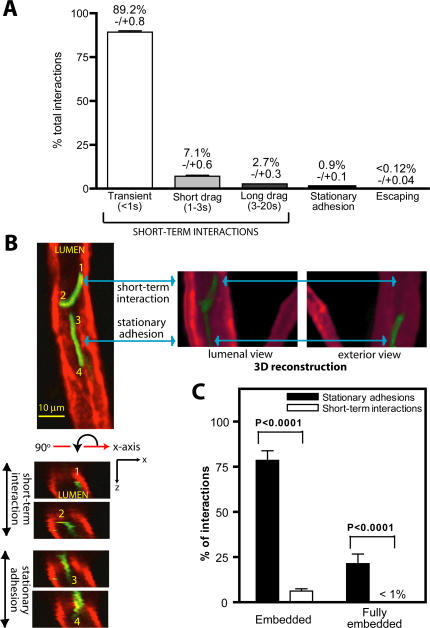
Visualization of *B. burgdorferi* short term interactions and stationary adhesions in postcapillary venules. A) Graphical summary of the distribution of different types of *B. burgdorferi* interactions in postcapillary venules of the skin microvasculature. The percentage of interacting spirochetes escaping from blood vessels is indicated in the last column. Interactions were scored from video footage of conventional epifluorescence IVM. A total of 10,920 spirochete interactions in 174 venules from 30 mice were analyzed. Standard error values are indicated above each column in the graph. Sample footage of the videos used to measure spirochete interactions is presented in Video S4. B) 3D spinning disk confocal IVM analysis of two green fluorescent spirochetes within a single venule: the upper spirochete is a short-term interaction and the lower one is a stationary adhesion. Endothelial cells were labeled with Alexa Fluor 555-conjugated antibody to PECAM-1 as noted in Fig. 2. Blood flow direction is upward. The top left micrograph is a view of spirochetes in the xy plane. Micrographs below the xy view show the bacteria and blood vessel in the xz plane, reconstructed from a series of z slices. Each micrograph in the xz series indicates the xz view of a single position along the length of each spirochete (positions labeled 1–4). To the right of the xy panel are lumenal and exterior views of a 3D reconstruction of the z-series. A rotating view.of this vessel reconstructed in 3D is shown in Video S5. C) Graphical summary of the average percentages of short-term interactions versus stationary adhesions that are partially or fully embedded in the anti-PECAM-1-stained vascular region. One hundred seventy-nine stable adhesions and 896 short-term interactions from z-series obtained from 29 vessels in 10 mice were analyzed. Standard error values are represented as bars above each column. Sample footage of short-term interactions and stationary adhesions visualized by spinning disk confocal IVM is shown in Video S3.

Quantitative analysis of *B. burgdorferi* interactions in postcapillary venules (where interaction rates could be most accurately quantified) revealed two major types of associations: short-term interactions and stationary adhesions (**Fig. 3A**). Associations were characterized and quantified in venules because interactions in larger veins were too numerous for accurate quantification and blood flow in capillaries could be blocked by trapped spirochetes. Interactions were quantified using conventional video-based epifluorescence IVM, which captures rapid adhesion events more effectively than spinning disk confocal IVM. All spirochetes that paused and associated, even briefly, with the vessel wall were counted, and the length of time required to travel 100 µm along the vessel wall was measured. *B. burgdorferi* that did not associate with vessels moved very rapidly, and were visible only as blurs; therefore, non-interacting spirochetes could not be quantified. However, the total number of *B. burgdorferi* in the bloodstream could be quantified by counting the number of spirochetes in blood samples using cell-counting chambers and was similar in all experiments.

Short-term interactions included two sub-groups: transient and dragging interactions. Transient interactions, which constituted the majority of interactions, were defined as those where *B. burgdorferi* slowed, associated briefly with the endothelium then detached in a tethering-type interaction cycle (for examples, see **Videos S3** and **S4**). Transiently associating spirochetes took less than 1 second to travel 100 µm in the vessel, but moved at least 8 times more slowly than the speed of blood flow, and frequently interacted only partially with the endothelium. Transient interactions could occur at the tip of the bacterium or elsewhere on the bacterial cell body, implying that the ends of *B. burgdorferi* are not the exclusive sites of tethering.

Transiently associating spirochetes that interacted with the endothelium along much of their length often slowed further and began dragging or crawling along the vessel wall. Short- and long-drag interactions were those in which spirochetes took 1–3s and 3–20s, respectively, to travel 100 µm along the vessel wall (for an example, see **Video S4**). *B. burgdorferi* dragging along the endothelium frequently slowed and stopped before dragging further along the wall in the direction of blood flow. The crawling movement observed at this stage of interaction may be similar to the crawling motion described for *Leptospira*, which results from translational motility in the context of simultaneous tethering of the spirochete at multiple distinct interaction sites [37].

Spirochetes that remained stationary at a single position on the vessel wall and did not translate along the vessel for at least 20 seconds were defined as stationary adhesions (for an example, see **Video S3**). Once stationary, these bacteria usually remained in the same place for at least two minutes (average: 10 minutes), and were aligned lengthwise along the vessel wall in the direction of blood flow. One end of stationary adhesions was usually less adherent and more mobile than the other, and sometimes exhibited a probing-type behavior that was more consistent with active gyration of the free spirochete end than with a passive rearrangement due to blood flow. The most stably adhered end was always pointing in the opposite direction to blood flow.

### Three-dimensional visualization of spirochetes interacting with the host microvasculature

To more closely examine *B. burgdorferi* interactions with the host microvasculature we performed 3D reconstruction on *z*-series micrographs of spirochetes and PECAM-1-stained vessels obtained used spinning disk confocal IVM. PECAM-1 is expressed on endothelial cells and concentrates at endothelial cell junctions [38], making it useful for visualizing both the lumenal endothelial surface of vessels, as well as the more PECAM-1-intensive intercellular junctions. PECAM-1 was visualized using an Alexa Fluor 555-conjugated antibody to PECAM-1 that has been used previously to study junctional extravasation of leukocytes *in vivo* [39].

Three-dimensional visualization of short-term interactions and stationary adhesions in venules revealed that these two classes of associating spirochetes differed with respect to their position relative to the PECAM-1-stained endothelium (**Fig. 3B** and **Video S5**). Greater than 93% of short-term interactions were localized on the lumenal surface of the vessel wall and were not observed to project into the PECAM-1-stained endothelium in three dimensional reconstructions (see positions 1 and 2 of the dragging spirochete in **Fig. 3B** lower and side panels). In contrast, a large majority of stationary adhesions (79%, **Fig. 3C**) were embedded in the PECAM-1-stained endothelium, either partially (57%) or along their entire length (21%) (**Fig. 3C** **;** see positions 3 and 4 of the stationary spirochete in **Fig. 3B** lower and side panels). **Video S5** presents a reconstructed three-dimensional view of a typical short-term interaction and stationary adhesion in a venule, with the short-term interaction visible only on the lumenal surface of the endothelium, and the adhering spirochete visible in both the lumen and projecting through the PECAM-1. The right hand panel of **Fig. 3B** shows lumenal and exterior views of these spirochetes from a 3D reconstruction. Because the PECAM-1 antibody stains the lumenal surfaces of endothelial cells, these data indicate that stationary adhesions are embedded more deeply in the endothelium than transiently interacting spirochetes, but do not imply that stationary adhesions project beyond the external boundary of vessels.

Also of interest, in the majority of stationary adhesions (71%), one end of the bacterium projected further into the PECAM-1 than the other end. The most deeply embedded portion of the adhesion was usually the most stable, since more superficially attached regions of the spirochete exhibited a greater range of movement (see **Video S3** for an example). This observation suggested that stationary adhesions might be slowly extravasating through the endothelium via the more deeply embedded tip. However, we did not detect any consistent outward migration of stationary adhesions during the experimental time period studied (up to 45 minutes), although it remains possible that such emigration might take much longer to occur.

**Figure 4:**
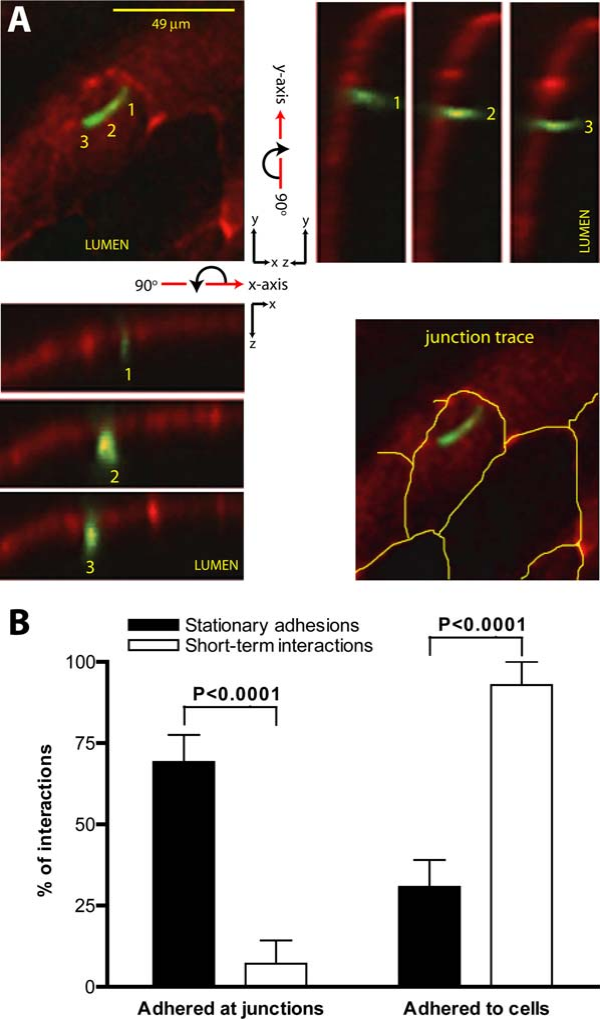
Characterization of *B. burgdorferi* interaction sites in postcapillary venules. A) 3D micrographs of a fluorescent spirochete adhered to an endothelial cell. Each micrograph in the yz and xz series indicates the yz or xz view of a single position along the length of the spirochete (positions labeled 1–3). Blood flow direction is down and to the left. A junction trace diagram in which junctions are indicated by yellow lines is provided in the bottom right panel to facilitate interpretation of the unmarked xy panel. B) Graphical summary of the average percentage of stationary adhesions or short-term interactions that are adhered at junctions, or adhered to cells. Eighty-two stable adhesions and 392 short-term interactions from z-series obtained from 20 vessels in 10 mice were analyzed. Standard error values are represented as bars above each column.

Finally, the localization of endothelium-interacting spirochetes was more precisely determined by examining the position of these interactions with respect to endothelial junctions (which are stained more intensely by anti-PECAM-1 antibody than the non-junctional surface of endothelial cells) (**Fig. 4**). To determine if PECAM-1 redistribution occurred in response to *B. burgdorferi*, we visualized junctions using PECAM-1 antibody before and after injection of infectious spirochetes, and examined junctional staining patterns from 5–45 minutes after spirochete injection. During this time frame, we observed no PECAM-1 redistribution in 95% of venules examined (n = 20 venules in 5 mice). Since junctional staining was sometimes incomplete, localization of interacting spirochetes was assigned only when the junctional boundaries of endothelial cells in the area of interaction were clearly demarcated. For the majority of stationary adhesions (∼70%), the most stable, deeply embedded region of adhesion occurred at junctions. However, about 25% were adhered primarily to endothelial cells (**Fig. 4B**). In contrast, the vast majority of transient and dragging short term interactions (93%) occurred on the surface of cells, with only 7% being found at junctions. Since cell surfaces make the largest contribution to total endothelial surface area, short-term interaction with cells may be a stochastic event, whereas stationary adhesion to junctions is likely the result of preferential localization.

### Four-dimensional visualization of spirochetes escaping from the host microvasculature

**Figure 5:**
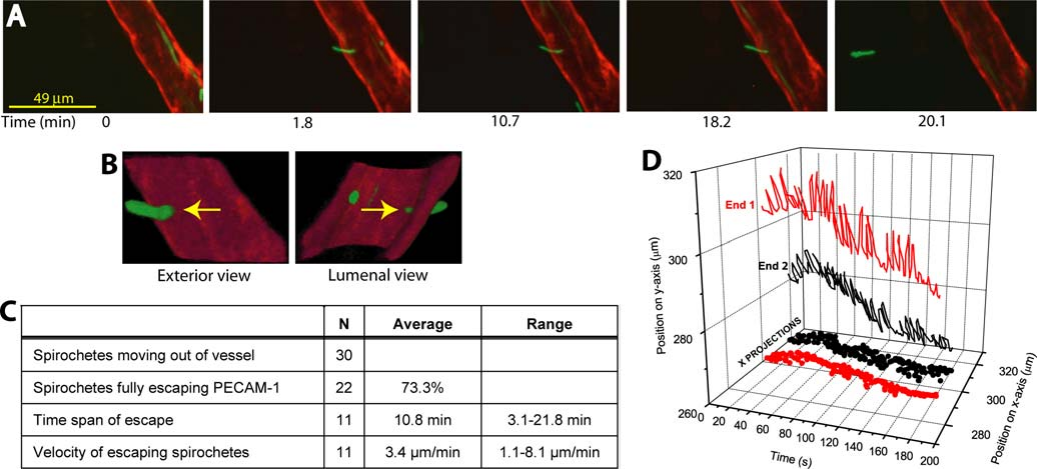
Visualization of *B. burgdorferi* escaping from postcapillary venules. A) Time lapse series of a spirochete escaping end-first from a postcapillary venule. Blood flow direction is up and to the left. Micrographs are projection views of z-series captured at the indicated time points. Exterior and lumenal 3D reconstruction views of the escaping spirochete (indicated by yellow arrows) at 1.8 minutes after the start of the time course are shown in B). C) Tabular summary of time span and velocity of escape for spirochetes visualized emigrating from postcapillary venules. Data were derived from analysis of 3D time lapse series obtained for a total of 30 emigrating spirochetes in 8 venules from 5 mice (emigration was determined by analyzing the direction of net spirochete displacement over time). Twenty-two of 30 spirochetes analyzed eventually fully escaped the PECAM-1-stained region, confirming that this outward movement constituted extravasation. Time span and velocity of escape were calculated for 11 spirochetes for which the start and endpoints of escape could be determined. The time span and velocity of escape for the remaining 19 spirochetes could not be calculated because either the start or end point of emigration was unknown. D) Graphical representation of reciprocal translational movement in a spirochete escaping from a postcapillary venule. The trajectories of each end of the spirochete (indicated by labels 1 and 2) are plotted. The total displacement and displacement velocities of this spirochete in the indicated time period were 19.7 µm and 6.6 µm/min, respectively, but the spirochete traveled a total of 610 µm as a result of reciprocal translation. The video footage from which this trajectory mapping was obtained is presented in Video S7.

*B. burgdorferi* escaped the microvasculature in an end-first fashion, and therefore projected out of the planes of view where most interactions were observed; thus, 2D visualization alone was insufficient for unambiguous analysis of this final stage of dissemination. Using 4D spinning disk confocal IVM (3D time courses), we measured the percentage of each spirochete's length that projected beyond the PECAM-1-labeled endothelium in successive z-series, and calculated the time span and speed of escape (**Fig. 5A–C**). As for stationary adhesions, most escaping spirochetes (83%) extravasated through endothelial junctions. Transmigrating spirochetes preceded by stationary adhesion were not observed in this study, although the time necessary to acquire successive z-series in 4D IVM might have precluded detection of short-lived adhesions that began extravasating. Escape took an average of 10.8 minutes, at an average net displacement velocity of 3.4 µm/min (**Fig. 5c**). The initial and final stages of the escape process were too rapid to capture visually in 4D, since they were faster than the 1–2 minutes necessary to acquire individual z-series (for sample 2D footage of the final stage of escape, see **Video S6**). Little net displacement occurred during the longer middle phase of escape, even though many bacteria in this phase exhibited obvious reciprocal translational motility (**Fig. 5D** and **Video S7)**. Reciprocally translating spirochetes could move in either direction as quickly as 624 µm/min, a speed which greatly exceeded their net displacement velocity. The great speed of these bacteria *in situ* might thus have accounted for our difficulty in capturing the initial and final stages of extravasation. The abridged timelapse shown in **Fig. 5A** illustrates the typical triphasic escape dynamic. In this case, 41% and 43% of the spirochete length passed out of the PECAM-1-stained endothelium in the first and last 2 minutes of extravasation, respectively, whereas only 16% of the spirochete traversed the PECAM-1 layer in the intervening 16 minutes. It was unlikely that the speeds of the initial and final stages of extravasation were the result of passive drifting of the bacteria through the endothelium, leading us to conclude that transmigration was largely driven by spirochete motility. Furthermore, the speed of the final escape phase, in which spirochetes appeared to burst away from the vessel (**Video S6**), suggested that the reciprocal translational motility observed in the middle phase was the result of partial adhesion of either the middle or the lagging portion of the spirochete to the endothelium [37]. Together, these observations suggested a prominent role for spirochete motility in the final stage of dissemination.

## Discussion

### Real-time visualization of *B. burgdorferi* interactions with the host vasculature

In this work technological advances in confocal microscopy have been coupled with intravital imaging methodologies to allow for the first time, high resolution, three and four dimensional, real-time visualization of the interaction of a bacterial pathogen with its living host. We have used this technology to study the interaction of the Lyme borreliosis spirochete *B. burgdorferi* with the microvasculature of one of its natural hosts.

**Figure 6:**
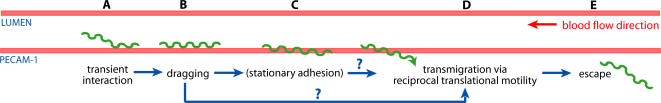
Model of the stages of vascular escape by disseminating *B. burgdorferi*. The PECAM-1-expressing surface of the endothelium is shown in red, and the position of the lumen is indicated. Initially, spirochetes transiently tether, then drag over the endothelial surface along their entire length. “Stationary” adhesions align lengthwise in the same direction as blood flow, and are at least partially “embedded” in the PECAM-1 region of vessels, with one end being more stably and deeply embedded than the other. Transmigrating *B. burgdorferi* escape the vessel end-first, a process which may be driven by translational motility. It is unknown if stationary adhesions represent an obligate step in the progression toward escape, or if they fulfill other biological functions.

One of the central events in the development of spirochetal diseases is hematogenous dissemination [40]. Previous investigations of dissemination by pathogenic *B. burgdorferi* were performed in a static environment using endothelial cell monolayers incubated with *B. burgdorferi* for several hours or longer, and methodologies that precluded direct observation of spirochete behavior [14], [15], [17]–[19]. In contrast, dynamic, 3D and 4D analyses of interactions in a living host under shear stress conditions indicate that *B. burgdorferi* escape from the microvasculature is a multi-stage process (as summarized in **Fig. 6**).

Spirochetes first transiently tether to the endothelium, usually at cell surfaces and not intercellular junctions (**Fig. 6A**), then drag and crawl along the vessel wall while interacting with the endothelium along much of their length (**Fig. 6B**). In contrast, stationary adhesions are usually established, at intercellular junctions (**Fig. 6C**), which also appear to be the major site for *B. burgdorferi* extravasation (**Fig. 6D**). Both stationary adhesion and extravasation may, therefore, be mediated by host and spirochete molecules distinct from those involved in short-term interactions. It remains unclear if stationary adhesions represent an obligate step in the progression toward vascular escape, or if they act as facilitators of this event by modifying the endothelium (see below). Similarly, multiple types of interactions are observed during leukocyte trafficking under the shear stress conditions of blood flow, which depends on a progressive association between different classes of endothelial and leukocyte molecules as the interacting cell slows down and locates an extravasation site [41]. It is probable that *B. burgdorferi* interactions with, and escape from the endothelium entail a similar progression.

Previous reports indicate that *B. burgdorferi* invade cultured endothelial cells by both intracellular and intercellular routes [14],[15],[18]. *Treponema pallidum* generally migrate through endothelial monolayers via intercellular junctions, whereas *Leptospira* primarily invade endothelial cells themselves; however, these spirochetes have also been observed in endothelial cells and junctions, respectively [12],[13]. Our results indicate that although the major extravasation route of *B. burgdorferi in vivo* is the intercellular junctions, a small percentage can also emigrate through endothelial cells. This conclusion raises the interesting possibility that other spirochetes could also exhibit the same versatile invasive capacity *in vivo*.

*B. burgdorferi* are known to interact with multiple host molecules that could mediate interaction with and invasion of the host endothelium *in vivo*; these include fibronectin, plasminogen, glycosaminoglycans, and integrins such as the vitronectin and fibronectin receptors [18], [42]–[47]. Indeed, we are currently using the technology described here to further study *B. burgdorferi* adhesion and have thus far identified several of the host and bacterial molecules involved at specific steps in the adhesion process (manuscript in preparation). These observations support a progressive model of spirochete adhesion under shear stress conditions in which different classes of host and *B. burgdorferi* proteins mediate distinct phases of interaction.

One of the most interesting, unprecedented and difficult findings to interpret in our study was that stationary adhesions projected deep into and sometimes through the PECAM-1-stained region of vessels, a phenomenon we refer to as “embedding.” Embedding could occur along the entire length of the spirochete, or at one end only. Interestingly, we found that *B. burgdorferi* embedded in the PECAM-1 region along their entire length adhered for much longer periods than partially embedded bacteria, and were frequently observed protruding through both sides of the PECAM-1 signal (e.g. see the stationary spirochete in **Fig. 3B**, lower panel), suggesting that they had migrated more deeply into junctions or endothelial cells than partially embedded adhesions. This observation may be consistent with the results of early electron microscopy studies demonstrating that *B. burgdorferi* can invade or be taken up by endothelial cells in monolayer cultures [15],[19], and is intriguing in light of previous proposals that spirochete evasion of the host immune system is mediated by “seeding” bacteria that escape immune surveillance in physically protected sites (reviewed in [48]).

Endothelial cells can be as thin as 0.1 µm [49] and the PECAM-1 antibody used in this study stained a 3 µm-thick region of the vessel wall. The observation that stationary adhesions often project beyond the PECAM-1-stained region suggests the possibility that these spirochetes are invading junctions or endothelial cells. However, the measured thickness of the PECAM-1 signal may overestimate the dimensions of the endothelium due to motion artifacts caused by respiration of the immobilized mouse. Therefore, we can only conclude that the apparently embedded state of stationary adhesions results from more intimate adhesion to the endothelium than that observed for short-term interactions. Additional higher resolution studies of stationary adhesions, performed under shear stress conditions, will be required to shed light on the true position of the spirochetes relative to the endothelium and the intriguing possibility that stationary adhesion of spirochetes might provide a protective mechanism for evasion of the immune response.

### Real-time visualization of *B. burgdorferi* escape from the host vasculature

Spirochete escape from the microvasculature was a rare event, even after intravenous inoculation with large doses of *B. burgdorferi*. Three dimensional timelapse data captured for 30 emigrating spirochetes revealed that *B. burgdorferi* escaping the microvasculature traversed the vessel wall end-first (**Fig. 6D**). Interestingly, multiple emigrating spirochetes were sometimes observed in the same vessel (data not shown). It appears unlikely that cases of multiple escape were the result of endothelial activation in response to *B. burgdorferi*, since these could be observed immediately after intravenous injection of spirochetes. Another possibility is that the presence of nearby stationary adhesions facilitated transmigration, since these adhesions were more abundant in vessels with escaping spirochetes, and as the site of transmigration was frequently in close proximity to a stationary adhesion (data not shown). Stationary adhesions adjacent to escape sites might modify their immediate vascular environment to promote emigration of other spirochetes.

Spirochetes emigrating end-first frequently exhibited a reciprocal translational form of movement that might drive much of the escape process. The average displacement velocity of emigrating spirochetes was 4-fold less than the average displacement velocity of *B. burgdorferi* translating in the extravascular tissues of the ear, suggesting that the endothelium presents significant physical barriers to transmigration. This conclusion is supported by the observation that the middle phase of escape was very slow relative to early and late stages. Previous work with *Leptospira in vitro* indicates that even cells adhered to immobile surfaces at a single point can move in a reciprocating fashion referred to as “staple movement”, likely as a result of a rapid (11 µm/s) lateral displacement of the adhesion site within the spirochete outer membrane [37],[50]. Such a model predicts that disruption of the adhesion site would cause a rapid change in the motile behavior of spirochetes, which is consistent with our observation that escaping *B. burgdorferi* “burst” out of the endothelium after a protracted period of reciprocal motility.

Early studies of *B. burgdorferi*, *T. pallidum* and *Leptospira* transmigration performed with endothelial monolayers found that non-motile spirochetes could not invade endothelium [12]–[17]. This conclusion is supported by the real-time imaging data reported in this study. It will, therefore, be important to directly examine the role of spirochete motility in emigration by coupling the use of the well-characterized *B. burgdorferi* motility and chemotaxis mutants [35], [51]–[53] with the technology reported here.

### Real-time high resolution 3D imaging – a new tool for studying bacterial host-pathogen interactions

We found that live fluorescent spirochetes can easily be observed *in situ* in living mice one month after subcutaneous and peritoneal inoculation. Furthermore, intravital microscopy can be performed in many of the tissues targeted by *B. burgdorferi* and other spirochetes, including the brain, liver, lung and joint cartilage [25]. It is, therefore, clear that the methodology described here could be a powerful tool for addressing a broad range of questions about host-pathogen interactions.

In addition to the types of experiments reported here, this methodology could be exploited for the study of a variety of bacterial pathogens in terms of their invasion of the vascular system, interactions with cellular components of the innate and acquired immune responses, for monitoring gene expression and migration patterns in different tissues over the course of infection and for analysis of chemotactic behavior in the host. The methodology may also be useful for monitoring events that occur immediately after needle or tick bite inoculation, routes of spirochete entry that more closely recapitulate the natural infection process than the intravenous injection of high numbers of blood adapted spirochetes.

In summary, dynamic and high resolution three-dimensional analyses of *B. burgdorferi* behavior in a living host have revealed numerous previously unobserved aspects of spirochete interaction with, and escape from, the host vasculature. The application of this powerful approach to the study of other micro-organisms is certain to enhance our understanding of the broad and always unpredictable repertoire of pathogenic agents and their interactions with their living hosts.

## Materials and Methods

### Construction of GFP expression plasmid pTM61

The terminator sequences (T1 x 4), *rbs*, *B. burgdorferi flaB* promoter and GFP coding sequences from pCE320(*gfp*)-P*_flaB_* [20] were PCR-amplified with flanking *Sac*I and *Kpn*I sites, using primers B696 (5′-ccggagctcatgataagctgtcaaacatgag-3′) and B697 (5′-ccggtacctcagatctatttgtatagttcatc-3′), and cloned into pCR Blunt II-TOPO (Invitrogen Canada, Burlington, ON) with the insert *Sac*I site proximal to the vector *Pst*I site, to make plasmid pTM41. This insert could not be cloned into the gentamycin-resistant version of the pBSV2 shuttle vector (pBSV2G) [54], presumably because replication origins and copy number sometimes affect the expression and toxicity of fluorescent proteins in *E. coli.* Therefore, a modified shuttle vector, pTM49, was constructed, in which the *colEI ori* of pBSV2G was removed by restriction digestion with enzymes *Mlu*I and *SnaB*I, and replaced with an *Mlu*I/*SnaB*I fragment from pCR Blunt II-TOPO containing the *pUC ori*. The (T1 x 4)-*P_flaB_-gfp* cassette from pTM41 was cloned into the *Sac*I/*Kpn*I sites of pTM49 to generate pTM61.

### *B. burgdorferi* transformations and screening

All strains were grown in BSK-II medium prepared in-house [55]. Electrocompetent infectious *B. burgdorferi* strain B31 5A4 NP1 [56] and non-infectious strain B31-A [57] (both B31-derived) were prepared as described [58]. Liquid plating transformations were performed with 50 µg pTM61 in the presence of 100 µg/ml gentamycin as described [59],[60]. Gentamycin-resistant *B. burgdorferi* clones were screened for: 1) the presence of *aacC1* sequences by colony screening PCR performed with primers B348 and B349 as described [61]; and 2) GFP expression by conventional epifluorescence microscopy. The presence of the pTM61 plasmid in non-integrated form in fluorescent strains was confirmed by agarose gel electrophoresis of total genomic DNA prepared on a small scale as described [62]. PCR screening for native plasmid content was performed as described [61],[63] and indicated that one fluorescent infectious *B. burgdorferi* clone (GCB726) contained all endogenous plasmids except cp9, which was displaced by the cp9-based pTM61 construct. Non-infectious strain GCB705 was used for experiments with non-infectious *B. burgdorferi*. PCR screening for native plasmid content indicated that GCB705 contained the same plasmids as the B31-A parent [61] (lp17, lp28-2, lp28-3, lp38, lp54, lp56, cp26, cp32-1, cp32-2/7, cp32-3 and cp32-9, but not lp21, lp25, lp28-1, lp28-4, lp36, cp9, cp32-6 or cp32-8). Plasmids lp25, lp28-1 and lp36 are known to be essential for infectivity [63],[64].

### Infectivity and plasmid stability studies

All animal studies were carried out in accordance with approved protocols from the University of Calgary Animal Research Centre. C3H/HeN (Harlan, Indianapolis, IN) and C57 BL/6 (Jackson Laboratory, Bar Harbor, ME) mice were infected by both intraperitoneal (5×10^4^ cells/ml) and subcutaneous (5×10^3^ cells/ml) needle inoculation. Ear punches and flank skin samples were cultured in Barbour-Stoenner-Kelly II (BSK-II) medium supplemented with 6% rabbit serum (Cedarlane Laboratories Ltd., Burlington, ON) with or without 100 µg/ml gentamycin. The percentage of *ex vivo* spirochetes that continued to express robust levels of GFP was calculated by counting the number of fluorescent spirochetes at 100 ms exposures compared to the number detected by phase contrast visualization.

### Preparation of fluorescent *B. burgdorferi* for direct bloodstream injection

For each experiment, infectious or non-infectious strains expressing GFP were freshly inoculated from glycerol stocks into 15 ml BSK-II medium containing 6% rabbit serum and 100 µg/ml gentamycin. *B. burgdorferi* were grown to 5×10^7^/ml, then diluted to 1–2×10^6^/ml in BSK-II medium containing 6% rabbit serum, 100 µg/ml gentamycin, 1× *Borrelia* antibiotic mixture (20 µg/ml phosphomycin, 50 µg/ml rifampicin and 2.5 µg/ml amphotericin B, prepared from individual antibiotics obtained from Sigma) and 1% C57 BL/6 mouse blood. Spirochetes were grown in the mouse blood for 48 hours at 35°C to a final density of ∼5×10^7^/ml. *B. burgdorferi* were pelleted (6,000×g for 15 min at 4°C), washed twice in PBS (Invitrogen Canada, Burlington, ON), and resuspended to 2×10^9^ *B. burgdorferi*/ml in PBS. All experiments were performed at a final density of ∼1×10^7^ spirochetes/ml of blood to facilitate quantitative analysis of interactions.

### Intravital microscopy of the ear

Animals were anaesthetized by intraperitoneal injection of a mixture of 10 mg/kg xylazine hydrochloride (MTC Pharmaceuticals, Cambridge, ON) and 200 mg/kg ketamine hydrochloride (Rogar/STB, London, ON). As previously described [65], a depilatory solution (Nair; Armkel LLC) was applied to the dorsal and ventral surfaces of the ear. After 10 min, the solution was gently removed using 0.9% normal saline and cotton swabs. The ear was mounted against the adjustable plexiglass microscope pedestal and held in place under a coverslip. Mouse rectal temperature was monitored via rectal thermometer and maintained at 37°C using a self-regulating heating mat.

### Intravital microscopy of the flank skin

The microcirculation of the ventral abdominal skin was prepared for microscopy as previously described [66]. Mice were anaesthetized and body temperature was monitored as described above. Briefly, after shaving a midline abdominal incision was made extending from the pelvic region up to the level of the clavicle. The skin was separated from the underlying tissue, remaining attached laterally to ensure the blood supply remained intact. The area of skin was then extended over a viewing pedestal and secured along the edges using 4.0 sutures. The loose connective tissue lying on top of the dermal microvasculature was carefully removed by dissection under an operating microscope. The exposed dermal microvasculature was immersed in isotonic saline and covered with a coverslip held in place with vacuum grease. The right jugular vein was cannulated to administer additional anaesthetic and fluorescent dyes. To visualize *B. burgdorferi*-endothelial interactions, 4×10^8^ spirochetes in 200 µl of PBS were injected directly into the jugular or femoral veins of anaesthetized mice. Three to six dermal venules (15–45 µm in diameter) were selected in each experiment.

### Conventional epifluorescence microscopy of *B. burgdorferi* on slides

Conventional epifluorescence microscopy was performed with a Leica DM IRE2 inverted microscope (Leica Microsystems, Frankfurt, Germany) equipped with an Orca ER cooled CCD camera (Hamamatsu, McHenry IL), using a 63× oil immersion objective, a narrow band GFP filter (480 −/+ 10 nm excitation wavelength; 510 −/+ nm emission wavelength: Chroma Technology Corp, Rockingham, VT) and exposure times of 100 ms. Sixteen-bit images were acquired using OpenLab 5.0.2 (Improvision Inc., Lexington, MA), and exported images in .tiff format were converted to 8-bit, colorized using indexed color and cropped in Adobe Photoshop CS prior to export and conversion to CYMK mode in Adobe Illustrator CS (Adobe Systems Inc., San Jose, CA). Identical image capture and adjustment settings were used for all images.

### Conventional epifluorescence intravital microscopy

Conventional epifluorescence intravital microscopy was performed using a Zeiss Axioskop microscope equipped with a 40× Wetzlar water immersion lens (Carl Zeiss Canada Ltd., Toronto, ON). Manual focusing was used to ensure that spirochetes remained in the focal plane throughout recording. A video camera (HS model 5100; Panasonic, Osaka, Japan) was used to project the images onto a monitor, and the images were recorded at 29.97 fps for off-line video playback analysis using a videocassette recorder. VHS analogue videos of conventional IVM experiments were converted to digital format using Windows Movie Maker (Microsoft Corporation, Redmond WA), and converted to .swf format using Macromedia Flash Professional 8 (Macromedia Inc., San Francisco, CA) without altering frame rate or editing frame sequence. Leukocyte recruitment was monitored by rhodamine staining of leukocytes, as previously described [67].

### Spinning disk confocal intravital microscopy

Spinning disk confocal intravital microscopy [68] was performed using an Olympus BX51 (Olympus, Center Valley, PA) upright microscope equipped with a 20×/0.95 XLUM Plan Fl water immersion objective. The microscope was equipped with a confocal light path (WaveFx, Quorum, Guelph, ON) based on a modified Yokogawa CSU-10 head (Yokogawa Electric Corporation, Tokyo, Japan). Endothelial cells and junctions were labeled with a monoclonal anti-PECAM-1 antibody (Fitzgerald Industries International, Inc., Concord, MA), conjugated to Alexa Fluor 555 (Molecular Probes, Invitrogen Canada, Burlington, ON). One hundred µl of Alexa-conjugated anti-PECAM-1 were injected per mouse (50 µg/mouse). In some experiments, 50 µl of 5 mg/ml FITC-albumin in normal saline (Sigma-Aldrich Canada Ltd., Oakville, ON) was injected to visualize blood vessels (250 µg/mouse). Laser excitation at 488 and 561 nm (Cobalt, Stockholm, Sweden), was used in rapid succession and fluorescence in red and green channels was visualized with the appropriate long pass filters (Semrock, Rochester, NY). Emission wavelengths for red and green channels were 593 nm and 520 nm, respectively, and no overlapping signal was detected in either channel. Exposure time for both wavelengths was 168 ms. A 512×512 pixels back-thinned EMCCD camera (C9100-13, Hamamatsu, Bridgewater, NJ) was used for fluorescence detection. Volocity Acquisition software (Improvision Inc., Lexington, MA) was used to drive the confocal microscope. Sensitivity settings were 255 and 251 for red and green, respectively, and autocontrast was used. Images were captured at 16 bits/channel in RGB. For timelapse series, manual focusing was used to ensure that spirochetes remained in the focal plane throughout recording. Red and green channels were overlaid using brightest point settings before export in .tiff or .mov format. Overlaid GFP and Alexa Fluor 555 .tiff images exported from Volocity were cropped in Adobe Photoshop CS without manipulation of signal levels or contrast prior to export and conversion to CYMK mode in Adobe Illustrator CS. Exported .mov files were imported without editing directly into Macromedia Flash Professional 8 for labeling and export as .swf files.

### Three-dimensional visualization and quantitative analysis of *B. burgdorferi* interactions with the vasculature

*Z*-series were collected using spinning disk confocal IVM, with images captured in both red and green channels for each slice. All image acquisition settings were as described above, except where noted in the Specific Image Acquisition Settings section, below. The localization of *B. burgdorferi* relative to the lumen, endothelium, endothelial junctions and extravascular tissue was scored for each *z*-slice in the series, using *xy*, *xz* and *yz* images constructed from the z-section series using Volocity 4.0.2. Scoring was performed independently by two individuals. Three-dimensional volume rendering (voltex) reconstruction of spirochetes in venules was performed in Amira 4.1.1 (Mercury Computer Systems, Chelmsford, MA) using series of GFP and Alexa Fluor 555 .tiff images exported separately from Volocity. Alpha and gamma settings were 1, and GFP and Alexa Fluor sensitivities were, respectively, 30–170 and 30–225. Animated rotation views of 3D volume rendering were exported as .mpeg files prior to import and labeling in Macromedia Flash Professional 8.

### Specific Image Acquisition Settings

All images were acquired and processed as described in the preceding sections. All timelapse series were captured at 0.94 fps, and exported at 5 fps, except the timelapse presented in **Video S1**, which was captured at 5.9 fps and exported at 50 fps. Other parameters: **Fig. 1C**: 0.485 µm/pixel (x and y); **Fig. 2**: 0.485 µm/pixel (x and y); **Fig. 3B**: 37 z-slices (45.3 sec/series), 5 µm step size, 0.485 µm/pixel (x and y), 1 µm/pixel (z); **Fig. 4A**: 19 z-slices (23 sec/series), 1 µm step size, 0.485 µm/pixel (x and y), 1 µm/pixel (z); **Fig. 5A**: 81 z-slices/time point (71 sec/stack), 0.5 µm step size, 0.485 µm/pixel (x and y), 0.5 µm/pixel (z); **Video S1**: settings same as in Fig. 1C; **Video S2**: settings same as in Fig.2; **Video S3**: 0.485 µm/pixel (x and y); **Video S5**: settings same as in Fig. 3b; **Video S6**: 0.485 µm/pixel (x and y).

### Statistics

For quantitative analysis, average and standard error values for different variables were calculated and plotted graphically for all vessels from all mice using GraphPad Prism 4.03 (GraphPad Software, Inc., San Diego, CA). Statistical significance was calculated in GraphPad Prism using a two-tailed non-parametric t-test with a 95% confidence interval.

## Supporting Information

Text S1 Supplementary Discussion
(0.04 MB DOC)

Video S1 Spinning disk confocal IVM video footage of fluorescent *B. burgdorferi* moving in the ear of a living C57 mouse. Experimental conditions were as described in the Figure 1c legend and Materials and Methods. Elapsed time is shown at the top right, and the scale at the bottom left. The time lapse was recorded at 5.9 fps and exported to video at 50 fps.
(1.52 MB SWF)

Video S2 Spinning disk confocal IVM video footage of fluorescent *B. burgdorferi* in a capillary of the skin vasculature. The time-lapse series shown in Figure 2 was extracted from this video. Experimental conditions were as described in the Figure 2 legend and Materials and Methods. Elapsed time is shown at the top right, and the scale is shown at the bottom left. The time lapse was recorded at 0.94 fps and exported to video at 5 fps. Direction of blood flow in this vessel is unknown.
(1.24 MB SWF)

Video S3 Spinning disk confocal IVM video footage of fluorescent *B. burgdorferi* interacting with a postcapillary venule of the skin vasculature. The position of a stationary adhesion (see Figure 3) is indicated. Most spirochetes visible in this video are interacting transiently (interaction times appear shorter than in real time because the video time scale is compressed). Experimental conditions were as described in the Figure 2 legend and Materials and Methods. Elapsed time is shown at the top right and the scale at bottom left. The time lapse was recorded at 0.94 fps and exported to video at 5 fps. Direction of blood flow is up and to the left.
(2.04 MB SWF)

Video S4 Conventional epifluorescence IVM video footage of fluorescent *B. burgdorferi* interacting with a postcapillary venule of the skin vasculature. The video is shown in real time (time indicated at the bottom). Blood flow direction is to the left. Experimental conditions were as described in the Figure 2 legend and Experimental Procedures, except that vessels were not counterstained with antibody to PECAM-1, and intravital microscopy was performed using a conventional epifluorescence microscope, to generate the data shown in Figure 3a.
(1.94 MB SWF)

Video S5 Three-dimensional reconstruction of vessel and fluorescent spirochetes shown in Figure 3b. Volume rendering reconstruction and animation were performed on the z-series shown in Figure 3b using the Amira 4.1 software package. The positions of the short-term interaction and stationary adhesion described in Figure 3b are indicated. Experimental conditions were as described in the Figure 2 legend and Materials and Methods.
(3.11 MB SWF)

Video S6 Spinning disk confocal IVM video footage of a transmigrating spirochete in the final stage of escape. Elapsed time is shown at the top right, and the scale at the bottom left. The time lapse was recorded at 0.94 fps and exported to video at 5 fps.
(1.13 MB SWF)

Video S7 Spinning disk confocal IVM video footage of transmigrating fluorescent *B. burgdorferi* repetitively translating forward and backward in the wall of a postcapillary venule. Video footage of the escaping spirochete whose trajectory is plotted in Figure 5d. Elapsed time is shown at the top right, and the scale at the bottom left. The time lapse was recorded at 0.94 fps and exported to video at 5 fps. Blood flow direction is upward.
(1.64 MB SWF)
