# Supplementary material for: Real-Time High Resolution 3D Imaging of the Lyme Disease Spirochete Adhering to and Escaping from the Vasculature of a Living Host
Source: PLoS Pathog. 2008 Jun 20;4(6):e1000090. doi: 10.1371/journal.ppat.1000090 (PMC2408724; doi:10.1371/journal.ppat.1000090)
Supplement: Text S1 — Supplementary Discussion (0.04 MB DOC) [file ppat.1000090.s001.doc]

**SUPPLEMENTARY INFORMATION**

**Absence of endothelial activation following spirochete injection**

The acute inflammatory response is characterized by two phases of endothelial activation, Type 1 and Type 2 (reviewed in [1]). Both are characterized by increased blood flow, vascular leakiness and leukocyte recruitment; Type 2 activation sometimes causes coagulation. Type 1 activation occurs within minutes of exposure to pathogens or other stimulants, lasts for 10-20 minutes, and is not dependent on gene expression. Type 2 activation occurs hours after exposure to stimulants, is more sustained, and requires upregulated transcription and translation of inflammatory response genes, including those encoding IL-8, E-selectin, VCAM-1 and ICAM-1. Consistent with the known temporal parameters of endothelial activation, previous *in vitro* experiments indicate that addition of *B. burgdorferi* lysates to endothelial monolayers results in transcriptional upregulation of IL-8 after 60 minutes, and E-selectin and ICAM-1 after 90 minutes [2]; the earliest reported time at which upregulated protein expression is observed for IL-8 and E-selectin is 4 hours [3]. However, activation is not required for association of *B. burgdorferi* with endothelial monolayers [4].

Not surprisingly, given the time frame of our experiments (5-45 minutes after intravenous injection of *B. burgdorferi*), we found no evidence for endothelial activation. Specifically: 1) blood flow rates measured in postcapillary venules 45-60 minutes after spirochete injection were normal for skin (794 -/+ 90 µm/s or shear rates of 225-284/s) [5]; 2) hematocrit levels were unaffected up to two hours after intravenous inoculation; and 3) there was no evidence of leukocyte recruitment, as examined with or without rhodamine staining of leukocytes. Finally, comparison of interaction rates with time elapsed post-inoculation indicated that no significant change in spirochete interaction rates occurred over the 45 minute period studied (Spearman correlation coefficients of 0.1004, 0.1295 and -0.02662 for, transient, dragging and stationary adhesion interaction rates, respectively, as calculated after scoring 40,813 interactions in 228 venules from 41 mice). Collectively, these observations implied that endothelial activation had not yet occurred in the 45 minute timespan of our experiments, and that even if a more subtle, undetected form of activation were present, it did not affect the interaction of *B. burgdorferi* with postcapillary venules of the skin.

**REFERENCES**

1. Pober JS, Sessa WC (2007) Evolving functions of endothelial cells in inflammation. Nat Rev Immunol 7: 803-815.

2. Ebnet K, Brown KD, Siebenlist UK, Simon MM, Shaw S (1997) *Borrelia burgdorferi* activates nuclear factor-kB and is a potent inducer of chemokine and adhesion molecule gene expression in endothelial cells and fibroblasts. J Immunol 158: 3285-3292.

3. Burns MJ, Sellati TJ, Teng EI, Furie M (1997) Production of interleukin-8 (IL-8) by cultured endothelial cells in response to *Borrelia burgdorferi* occurs independently of secreted IL-1 and tumor necrosis factor alpha and is required for subsequent transendothelial migration of neutrophils. Infect Immun 65: 1217-1222.

4. Ma Y, Sturrock A, Weis JJ (1991) Intracellular localization of *Borrelia burgdorferi* within human endothelial cells. Infect Immun 59: 671-678.

5. Mitchell DJ, Li P, Reinhardt PH, Kubes P (2000) Importance of L-selectin-dependent leukocyte-leukocyte interactions in human whole blood. Blood 95: 2954-2959.
